# Editors Introduction to the Special Issue on Advances in Cognitive and Behaviour Therapies and Applications in Health Practices

**DOI:** 10.3390/jcm11185269

**Published:** 2022-09-07

**Authors:** Louise McHugh, Rhonda M. Merwin

**Affiliations:** 1 School of Psychology, Newman Building, University College Dublin, D04 V1W8 Dublin, Ireland; 2 Department of Psychiatry and Behavioral Sciences, School of Medicine, Duke University, Durham, NC 27708, USA

The last thirty years have seen significant advances in cognitive and behavioural psychotherapies and their application in health care/clinical practices. These include the integration of acceptance/mindfulness and neurobiological findings in theory and practice, greater orientation to processes of change, and the use of mobile technology and digital and just-in-time interventions. The current *Journal of Clinical Medicine* Special Issue on new Advances in Cognitive and Behavioural Psychotherapies aims to bring together empirical papers relevant to new advances in Cognitive Behaviour Therapy or theory that include clinical outcome studies, studies of the dynamic interplay of influential factors (e.g., personalised medicine, processes of change research, lab-based research relevant to clinical interventions), and systematic literature reviews. To promote this line of work, an announcement for a Special Issue was posted early in 2021, at which point submissions were opened.

Twelve manuscripts were handled for this Special Issue by two guest editors (McHugh and Merwin). Eight manuscripts on this topic were chosen to include in this issue after rigorous peer review. Articles in the area of mindfulness-based interventions, acceptance and commitment therapy, and moral reasoning development are included. Health areas of body image, HIV, Inflammatory Bowel Disease, eating disorders, schizophrenia, the treatment of healthcare professionals, emotion regulation, and pain sensitization conditions were included. Six articles in the Special Issue are empirical studies with two RCTs and one with a single case experimental design, and there are two systematic reviews.

The first paper is focused on psychological flexibility—the most supported process of change in psychological interventions [1]. Psychological flexibility refers to the ability to be flexible, aware, and engaged in coping with one’s problems and is central to health and well-being [2]. The study examined whether untrained individuals could embody and detect psychological flexibility. The participants were photographed assuming a body posture that reflects when they are at their “best” and “worst” when dealing with a difficult psychological issue or a challenge. The photographs were then rated on the three dimensions of psychological flexibility: openness, awareness, and engagement. The participants included individuals from the United States and Iranian immigrants.

There was a significant difference in ratings by picture type, indicating that the participants naturally (without training) displayed more open, aware, and engaged postures when embodying themselves at their best at dealing with a problem and more closed, unaware, and unengaged postures when at their worst, and with high inter-rater reliability by untrained individuals. These findings suggest that people may learn psychological flexibility as an adaptive approach to problems (and can recognise it in others), even when it is not explicitly trained. It also highlights body posture as social communication and regulation, with possible implications for the health and well-being of close individuals [3] and the potential to use more objective indicators of key processes of change in psychological interventions to advance beyond self-report measures [1].

However, the authors also report another interesting finding of an interaction between the ratings and location, with the postures of Iranian men suggesting cross-cultural differences in the topography of effective coping. This highlights the importance of taking a multilevel approach to the study of human behaviour that considers the influence of progressively larger ecosystems [4]. In which the individual and their behaviour is situated [1].

The next five papers are empirical investigations of interventions. Interventions address an array of human problems, from the management of chronic disease to eating disorders and violence prevention. The first three papers focus on adapting Acceptance and Commitment Therapy (ACT) to a variety of health-related problems. Acceptance and Commitment Therapy (ACT) is a contemporary cognitive and behavioural therapy that is grounded in functional contextualism and is process-based [5]. Over 800 randomised controlled trials have been conducted [6] in the nearly forty years since the first study on ACT was published [7]. The papers included in this Special Issue describe the adaptations of ACT for HIV, Inflammatory Bowel Disease (IBD), and Eating Disorders (EDs); two engage technology in intervention delivery. We return to ACT in a systematic review on ACT for Central Pain Sensitization Syndromes (CPSS) at the end of this Special Issue. A fourth paper takes a different therapeutic approach, developing a Moral Reasoning Development Intervention to address violence in schizophrenia, and the final empirical paper uses Mindfulness-Based Stress Reduction (MBSR) to help the helper (the healthcare worker). Each paper has a different strength or point of interest.

The first of the five intervention papers describes the development of an ACT-based intervention for hospitalised, out-of-care persons with HIV who endorse avoidant coping. The goal of the intervention was to improve HIV treatment engagement, and the aim of the current study was to examine the initial feasibility and acceptability and modify the intervention based on key stakeholder feedback in preparation for a randomised controlled trial (RCT). The investigators learned what was more or less helpful, including an appreciation for the delivery settings and interventions that resonated with the participants. The participants that provided feedback (about 50% of the enrolled) indicated that they would recommend it to others. As a result of the pilot, the intervention was simplified with key concepts repeated, some graphics were changed for better representation and sensitivity to the patient population, and the intervention materials were translated into Spanish. This paper was unique in its target of health care utilisation/retention, but also in including patients in the research enterprise, which can be rare. It was also of interest in its focus on a potentially marginalised population.

The next paper uses Single Case Design (SCD) methods to help patients with Inflammatory Bowel Disease (IBD) cope with distress. In the first experiment, one therapeutic session was delivered face-to-face. The results of this study indicated that a higher dose was necessary. In the second experiment, a two-session protocol delivered by telehealth and supplemented with follow-up telephone calls was introduced. The findings indicated that this dose was significant in producing change: 50% of the patients experienced reduced stress, increased engagement in valued action, and increased functioning. This study illustrates how telehealth may be used to deliver more adequate doses with less burden than in-person interventions. It is also of interest in its use of SCD, underutilised in the development of psychological interventions despite advanced statistical techniques to test and quantify change.

The third ACT paper involved a Randomised Controlled Trial of a digital, gamified intervention for women and girls at high risk for developing an eating disorder. The entire intervention was delivered online. The participants followed an avatar (main character) through a storyline and learned ACT-based coping skills as the character faced challenging experiences related to her body weight and shape. The participants learned skills vicariously and through the completion of interactive exercises. The participants in the ACT group evidenced greater reductions in ED symptoms relative to the waitlist control group, suggesting that this may be a promising direction for early ED intervention. This study was unique in delivering a completely online intervention with high potential for wide dissemination and in the use of gamification to engage young people and train skills to reverse the trajectory of an at-risk population.

The next paper looked at an integrated Moral Reasoning Development Intervention (MRDI) for the management of repeated violence in schizophrenia. The intervention focused on the motivation of behaviour and behaviours guided by cognitive processes, moral and ethical judgments, and responsibility attribution, rather than impulsive or emotional responses. The participants practised moral reasoning skills in personalised scenarios. Unlike other interventions included in this systematic review, MRDI challenged cognitive distortions specifically related to intent, causes and consequences of behaviour. However, similar to ACT and other contemporary CBTs in this review, it reinforced cognitive flexibility and flexible perspective-taking. The MRDI protocol also taught anger management, conflict resolution, and social skills. MRDI outperformed treatment-as-usual in improving moral reasoning and decreasing violence. This study is an example of teaching individuals to consider their automatic assumptions and orient to broader personal values or goals in decision making, along with the broader dimension of self-regulation as a process of change. It is also an example of a personalised approach with in vivo practice.

A final empirical paper looked at Mindfulness-based stress reduction (MBSR). MBSR involves weekly group classes and daily mindfulness exercises to practise at home over an 8-week period. There are hundreds of studies demonstrating the efficacy of the 8-week programme [8]. The current study compared a standard length to an abbreviated Mindfulness-Based Stress Reduction program for anxiety and depression in healthcare professionals. The standard MBSR training program had a positive effect on anxiety and depression and promoted long-lasting effects in tutors and resident practitioners. These effects were not observed in the abbreviated program. This provides important information on dosage and attempts to shorten interventions. This study is also an example of interventions extending to practitioners—potentially redefining or eliminating somewhat arbitrary distinctions between “patients” and “practitioners”.

The final two papers are systematic reviews. The first reviews interventions on emotion regulation (ER). ER interventions improved ER as expected and were superior to waitlist or treatment as usual; however, there was limited evidence to suggest they were superior to other active treatments. Caution should be taken here, as the review also highlighted the need to further develop this area, with most studies having less than optimal methodological rigour (only three studies were rated as “good”, none were rated as “excellent”). The second systematic review summarised the empirical base on ACT for central pain sensitization syndromes (CPSS). There were 21 studies, of which four were high quality (others were moderate or poor). Overall, ACT appears to reduce subjective CPSS symptoms and improve quality of life, outperforming waitlist controls, psychoeducation, and pharmacological interventions.

We are delighted to have served as guest editors for such a diverse and exemplary set of manuscripts. We are grateful for the high quality of the work of not only the academics and authors but also of the JCM staff and reviewers who made this Special Issue possible. We believe that by bringing together these advances in this Special Issue, we have highlighted the important work that is ongoing in this area that is contributing to better practice and outcomes in clinical medicine. We hope you enjoy these articles as much as we did and that they inform your work.

## Data Availability

Not applicable.
